# Effective Assembly of Nano-Ceramic Materials for High and Anisotropic Thermal Conductivity in a Polymer Composite

**DOI:** 10.3390/polym9090413

**Published:** 2017-09-05

**Authors:** Haeleen Hong, Jong Uk Kim, Tae-il Kim

**Affiliations:** 1 School of Chemical Engineering, Sungkyunkwan University (SKKU), 2066 Seobu-ro Jangan-gu, Suwon 16419, Korea; kirin_h@nate.com (H.H.); kjo1687@gmail.com (J.U.K.); 2 Center for Neuroscience Imaging Research (CNIR), Institute for Basic Scienece (IBS), 2066 Seobu-ro Jangan-gu, Suwon 16419, Korea

**Keywords:** nanocomposites, thermally conductive composite, electrical insulating composite, ceramic-polymer composite, networked assembly, ceramic network

## Abstract

Recently, anisotropic heat dissipation and its management have drawn attention as a promising technique for highly integrated electrical devices. Among many potentially challenging materials such as carbon nanotube, graphene, metal particles, and inorganic ceramics commonly used for high thermally conductive fillers in a composite form, nanoscale ceramic fillers are considered ideal candidates due to their thermal conductivity, electrical insulation, and low thermal expansion coefficient. However, enhancing the thermal conductivity of a randomly dispersed ceramic-polymer composite is limited by its discontinuous filler contact and thermal expansion coefficient mismatch. Thus, recent research has focused on how to assemble and generate highly networked filler contacts to make effective pathways for heat flow, with minimized concentration of the filler in the composite. In this review, we will introduce several essential strategies to assemble fillers with a two- or three-dimensional networked composite for highly enhanced anisotropic heat dissipation. Moreover, this review elucidates filler alignment effects compared to randomly dispersed ceramic composites.

## 1. Introduction

**Figure 1:**
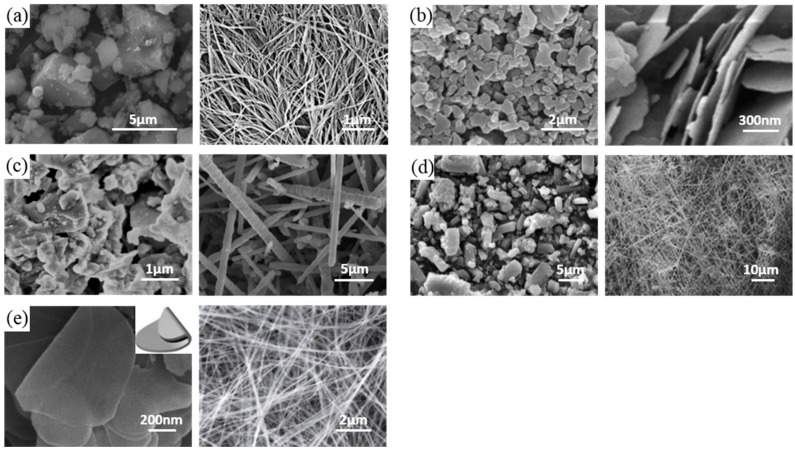
Examples of several high thermal conductive nanoscale ceramic fillers. (**a**) Aluminum nitride (AlN) spherical particles and nanowires [8,9]. (**b**) Aluminum oxide (Al_2_O_3_) spherical particles and platelets [10,11]. (**c**) Silicon carbide (SiC) particles and whiskers [12,13]. (**d**) Silicon nitride (Si_3_N_4_) spherical particles and rods [3,14]. (**e**) Boron nitride (BN) plates and nanotubes [4,15]. {(**a**)(left): Reprinted with permission from [8] Copyright (2015) Elsevier. (**a**)(right): Reprinted with permission from [9] Copyright (2009) Springer. (**b**)(left): Reprinted with permission from [10] Copyright (2014) Elsevier. (**b**)(right): Reprinted with permission from [11] Copyright (2010) Royal Society of Chemistry. (**c**)(left): Reprinted with permission from [12] Copyright (2008) Elsevier. (**c**)(right): Reprinted with permission from [13] Copyright (2012) Elsevier. (**d**)(left): Reprinted with permission from [3] Copyright (2007) Elsevier. (**d**)(right): Reprinted with permission from [14] Copyright (2013) Royal Society of Chemistry. (**e**)(left): Reprinted with permission from [4] Copyright (2016) John Wiley and Sons. (**e**)(right): Reprinted with permission from [15] Copyright (2013) John Wiley and Sons}.

With the dramatic miniaturization, functionalization, and integration of electronic devices on limited dimensions of a substrate for high-performance electronics, particularly for flexible electronics formed on a sheet of plastic, thermal management has received attention as one of the most challenging and promising techniques. Heat dissipation from electrical components such as bare chips and light emitting diodes obviously plays a critical role in maintaining device lifetime and reliability under high-performance operation [1,2]. Thus, thermal interfacial material (TIM), a heat sinking substrate, and a package are potentially applicable for many industrial uses. Moreover, mechanical properties such as flexibility, facile processability, electrical insulation, a low cost, and a light weight are critical. Usually, thermal conductive materials that satisfy all requirements mentioned above are composed of composite films with nanoscale fillers with high thermal conductivity and a polymeric matrix that can be easily processed with outstanding mechanical properties. Among various materials (Figure 1) that can be used as high thermal conductive materials, such as metals, carbon materials, and ceramics, ceramics have been considered ideal candidates for integrated electrical devices owing to their electrical insulating nature, high thermal conductivity, and low thermal expansion coefficient [3,4]. Many researchers have investigated ceramic-polymer composites for high thermal conductive composites. The most common technique of synthesizing ceramic-polymer composites is to randomly disperse ceramics in the polymer via solution mixing or milling because of its facile processing. However, enhancing the thermal conductivity of the disordered dispersion of fillers is limited due to the low probability of contact between fillers. These contacts are required for thermal conductivity because phonon heat conduction can occur at short distances between fillers [5]. Although many methods with high loading fillers have been introduced, the mechanical properties of composites deteriorate in efforts to surmount their low thermal conductivity.

To overcome these limitations, recent studies have aligned nanoscale ceramics in a particular direction or through the construction of 3D networks. High thermal conductivity can be achieved even in minimized loading concentration of fillers by forming 3D-networked fillers with a highly directional heat flow [6,7]. In addition, the reduction of the thermal expansion effect on electronic devices is observed because the thermal expansion is restricted to the direction of aligned fillers. In this review, we will present several kinds of ceramic fillers that are appropriate for high thermal conductive composites. We will also introduce effective assembly techniques to form aligned fillers. Effective ways of constructing 3D networks and enhancing or controlling heat flux through the composite are presented. In addition, we will evaluate recent works in terms of thermal conductivity and dielectric property, focusing on the effect of filler alignment. The effects of aligned and 3D networked ceramic fillers in a composite are also discussed in comparison with randomly dispersed filler composites.

## 2. Materials for Thermal and Electrical Properties of Nanoscale Ceramics

Up to date, many nanofillers such as metal particles, carbon materials, and ceramics embedded in the polymeric matrix have been demonstrated to be able to effectively dissipate heat due to their inherent high thermal conductivity [16,17,18,19,20]. However, metal- and carbon-based materials have superior electrical properties, too [16,21]. This is due to potential short circuits, which allow unintended electrical paths caused by the lack or very low impedance of carbon or other electrical conductive material. This limits the use of metal and carbon composites for applications in highly integrated electrical devices that need to avoid signal propagation or attenuation. Distinguished from others, polymer-based composites with nanoscale ceramic fillers have drawn attention because of their low dielectric permittivity, high electrical resistivity, high thermal conductivity, chemical inertia, and low thermal expansion [22,23].

Figure 1 shows several different materials made of nanoscale ceramics, such as aluminum nitride (AlN), alumina (Al_2_O_3_), silicon carbide (SiC), silicon nitride (Si_3_N_4_), and boron nitride (BN), which have been widely used as inorganic fillers in a polymer matrix to form a composite with polymers [24]. They are attractive ceramic fillers due to their superior thermal conductivity (30–390 W/mK), low dielectric constant (4.5–9 at 1 MHz), and high electrical resistivity (>1013 Ω cm) (Table 1). Although several examples with AlN have been presented, AlN has a chemical reaction with moisture in the air that generates Al(OH)_3_ and NH_3_ [25,26]. These undesired byproducts can damage electrodes and devices. Therefore, the industry is reluctant to use composites with AlN nanoceramics. Al_2_O_3_ has good chemical stability and relatively high cost-effectiveness, but the Al_2_O_3_ ceramic has much lower thermal conductivity (30–42 W/mK) than other ceramics, such as AlN or BN [27]. SiC is an attractive alternative candidate for high-temperature applications because of its high thermal conductivity and stability. However, it has limitations for some applications in highly integrated devices because it has too high a dielectric constant (40 at 1 MHz) compared to other ceramics (4.5–9 at 1 MHz) [28]. Si_3_N_4_ has a thermal conductivity lower than AlN or BN. However, it has a higher chemical stability, higher erosion resistance, and higher cost-effectiveness than both AlN and BN. This has been confirmed by many industrial applications [29,30]. Interestingly, unlike others, BN has an anisotropic heat flow due to its honeycomb molecular structure. When BN nanosheets are aligned parallel to the c-axis, the thermal conductivity is almost 20 times (600 W/mK) greater than that (30 W/mK) when BN nanosheets are aligned perpendicular to the c-axis [31,32]. Therefore, particular procedures are necessary for BN-nanocomposites to attain high thermal conductivity.

## 3. Assembly of Nanoceramics for Composite

### 3.1. Randomly Dispersed Polymer-Ceramic Composites

**Figure 2:**
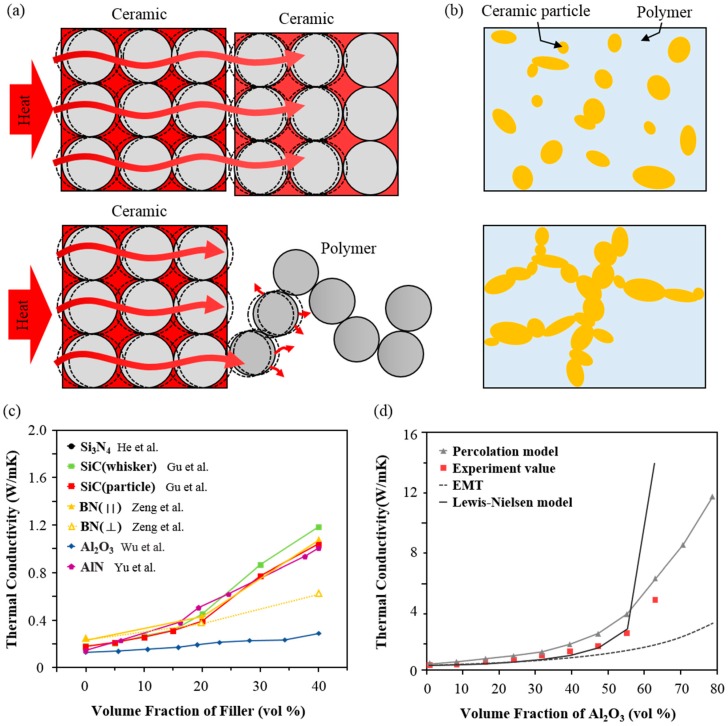
(**a**) The thermal conductive mechanism in ceramic-ceramic (upper) and ceramic-polymer (lower). (**b**) Scheme of a randomly dispersed ceramic-polymer composite with below (upper) and above (lower) the percolation threshold volume fraction of filler. (**c**) Comparison of thermal conductivities with randomly dispersed various composites and their volume ratio effects [3,22,35,45,50]. (**d**) Comparison between several simulated fittings of thermal conductivity based on theories and experimental values for randomly dispersed Al_2_O_3_ filler-polymer composite [51]. Reprinted with permission from [51] Copyright (2000) IEEE.

Polymers are commonly used materials with essential advantages such as a sufficient dielectric property, a low cost, facile processability, flexibility, and a light weight [40]. It is preferable to use a polymer as a composite matrix with ceramic nanofillers to allow for processability and flexibility [41,42]. Therefore, the technique that allows randomly dispersed fillers in the polymer is regarded as an efficient means of making the composite due to its facile nature, low cost, and mass productive process. Within the composite, ceramic fillers are generally in contact with the polymeric interface, and heat transfer between ceramic fillers is conducted directionally by atomic vibration while phonon scattering happens during the heat transfer from the ceramic filler to the polymer matrix due to the vibration and rotation of the polymer chain (Figure 2a) [43]. However, as percolation (limited contact probability between adjacent nanofillers) determines the thermal conductivity of composites, fillers are usually encapsulated with the polymer under the percolation threshold due to the poor interfacial affinity between ceramic fillers and the polymer. This can cause thermal resistance at the interface (Figure 2b) [44]. Figure 2c shows the experimental results of a heat transfer in randomly dispersed ceramic-polymer composites. As pristine polymer matrixes usually have a low thermal conductivity of around 0.2 W/mK, the ratio of ceramic fillers embedded in the polymer matrix is significant. When nanoscale fillers such as Si_3_N_4_, SiC, BN, Al_2_O_3_, and AlN are added to the polymer, the thermal conductivity is enhanced with an increasing amount of loading fillers. As shown in Figure 2c, each graph has a transition point at which the slope steeply increases, indicating the percolation threshold. However, thermal conductivities are very limited (up to about 1.5 W/mK) even at high concentrations of Si_3_N_4_ (40 vol %) (Figure 2c) [3]. 

To predict the thermal conductivity, several thermal conductive models have been proposed. According to effective medium theory (EMT), which assumes that fillers are completely surrounded by the polymer matrix, the thermal conductivity of randomly dispersed plate-like filler-polymer composites is increased proportionately with the volume ratio of the fillers [45]. Thermal conductivity (*K*) of the composite from EMT is determined as follows:

(1) $$K=K_{m}\frac{3+2V_{f}\left(\frac{K_{p}-K_{m}}{K_{m}}\right)}{3-V_{f}\left[\left(1-\alpha \right)-\frac{K_{m}}{K_{p}}\right]}$$

where *K*_p_ and *K*_m_ are the thermal conductivity of the filler and the matrix, respectively, and *V*_f_ is the volume fraction of the fillers. α is presented by $\left(R_{\mathrm{BD}}\cdot K_{m}\right)/d$, where *d* is the thickness of the fillers, and *R*_BD_ is the interfacial thermal resistance between the fillers and the matrix. The percolation model assumes that fillers can form heat conductive channels with increasing volume fractions of the filler so that the slope visibly shifts to steep at the percolation threshold [46]. Moreover, the Lewis-Nielsen model covers a wide range of filler shapes with relatively close results [44]. The thermal conductivity from the Lewis-Nielsen model is shown below:

(2) $$K=\frac{1+AB\phi }{1-B\psi \phi }$$

where A is the shape coefficient for the filler particles, ϕ is the filler’s volume fraction, B is presented by $\left(k_{1}/k_{m}-1\right)/\left(k_{1}/k_{m}+A\right)$, and ψ is $1+\left(1-\phi_{m}\right)\phi /\phi_{m}^{2}$. $\phi_{m}$ is the maximum filler volume fraction, which is influenced by the shape of the particle and the type of packing [19,44,47,48,49]. Figure 2d shows several models with experimental data of Al_2_O_3_ filler-polymer composite. At a low volume fraction of filler (up to 40 vol %), experimental data followed EMT and the Lewis-Nielsen model. However, with more than 50 vol % of filler, the Lewis-Nielsen model became unstable, and the experimental data tended to rise sharply, which showed a trend similar to that of the percolation model.

However, the randomly dispersed nanocomposite has huge hysteresis of thermal conductivities between in-plane and through-plane, as shown in comparisons of the BN orientation (‖) and (⊥) of Figure 2c. Contrary to conventional in-plane thermal conductivity of the nanocomposite, the through-plane thermal conductivity usually deteriorates by at least a few orders of magnitude. The reason is that anisotropic fillers such as plate, tube, and wires tend to arrange in horizontal directions rather than stochastically out-of-plane due to gravitational force or shear force when the composite film is generated [52].

Moreover, the loading concentration of fillers contributes to surmounting the increase in thermal conductivity. However, mechanical properties such as crack-resistance and processability of composites dramatically decline as loading concentration increases [53]. Consequently, challenging issues for high thermal conductivity composites consist of maximizing the loading concentration of nanoceramic fillers while maintaining the mechanical properties of the composite film, without compromising directional heat conductivity with the anisotropic shape of nanofillers.

### 3.2. Anisotropically Aligned Ceramic Fillers for a Composite

**Figure 3:**
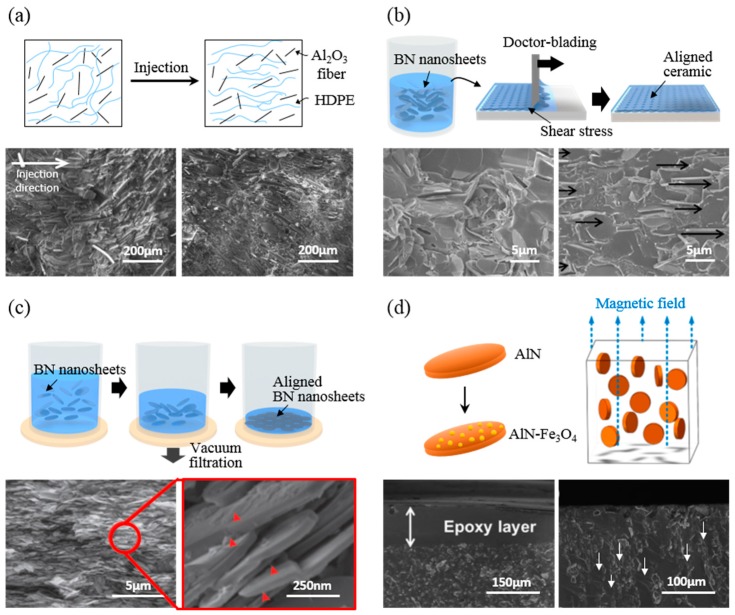
Schematic illustration and their SEM images of aligned ceramic fillers in a polymer matrix. (**a**) Schematic illustration for the orientation of Al_2_O_3_ fibers in high-density polyethylene (HDPE) formed by directional shear injection force and cross-sectional SEM images of the Al_2_O_3_ composite [23]. Reprinted with permission from [23] Copyright (2007) John Wiley and Sons. (**b**) Schematic illustration of the process for a BN/polyvinyl alcohol (PVA) composite film (20 vol % of BN) whereby shear stress of meniscus is applied with doctor blading, and its cross-sectional SEM images [56]. Reprinted with permission from [56] Copyright (2015) American Chemical Society. (**c**) Schematic illustration of the preparation of nacre-like BN/PVA composite paper (6 wt % of PVA) aligned by vacuum filtration force, and its cross-sectional SEM images [57]. Reprinted with permission from [57] Copyright (2015) Royal Society of Chemistry. (**d**) Anisotropic assembly of AlN pallets coated with Fe_3_O_4_. The surface modified filler is sensitive to a magnetic field. It is aligned as shown in the schematic. Cross-sectional SEM images of randomly dispersed AlN/epoxy without magnetic force (left) and AlN-Fe_3_O_4_/epoxy aligned by magnetic field (right) [53,58] are shown. {(**d**)(upper): Reprinted with permission from [53] Copyright (2016) Elsevier. (**d**)(bottom): Reprinted with permission from [58] Copyright (2015) American Chemical Society}.

Although ceramic materials and their nanofillers have inherently high thermal conductivity, it is difficult for the composite with randomly dispersed nanofillers to directly satisfy the practical requirement for a directional thermal dissipation layer due to its very limited thermal conductivity. A critical factor is that the interfacial thermal resistance between fillers in a polymer matrix could lead to phonon scattering caused by the lack of contact of each filler [54]. This is mainly caused by the limitation of thermal conductivity increase, even in a high concentration of fillers. Thus, effective percolation and alignment induced by optimized physical contacts between adjacent fillers in a polymer matrix can contribute to the generation of successful pathways for heat conduction because the shape of ceramic fillers (typically plate-like, rod-like, or fiber morphologies) is anisotropic. The construction of aligned filler networks can lead to high thermal conductivity in the orientation direction with a relatively low loading of fillers [55]. Here, we show several ways to align fillers, including injection molding [23], doctor blading [56], vacuum-assisted alignment [57], and electromagnetic field alignment [58] by external force such as shear force or magnetic force (Figure 3).

#### 3.2.1. Injection Molding

Injection molding is a typical method of obtaining anisotropically aligned fillers, especially in a fiber-shape template [59]. When a fluidic dispersion of anisotropic fillers and polymer is injected through a mold or a nozzle, the applied shear stress tends to cause fillers to align in the direction of injection, rather than perpendicular to the direction of injection [60]. Figure 3a shows the alignment of Al_2_O_3_ fibers (with an average diameter of 10 μm) in high-density polyethylene (HDPE). The thermal conductivity through the injected direction is enhanced by as much as 17.5% compared to that of a randomly dispersed case [23]. With the molding injection method, various types of molds can be used when needed. In addition, it is possible to orient fiber-like fillers in an in-plane or out-of-plane direction depending on the mold shape. However, in the case of a thin film, this technique of aligning fillers in an out-of-plane direction is less preferred. Moreover, when the flow of injection becomes larger, the orientation of the high filler fraction composite is almost random due to the influence of the interaction between fillers [48].

#### 3.2.2. Doctor Blading

Doctor blading is a fascinating technique of achieving a horizontal array of anisotropic fillers. Owing to its facile continuous process without any separate equipment, doctor blading is advantageous for mass production. It can be easily applied to actual industries. Because of the fluid flow involved in the doctor blading process, a strong field of shear stress occurs at the boundary of the blade, which affects the edge of the carrier sheet where it meets the blade. The shear force can align anisotropic fillers in the direction of flow [61,62,63,64]. The doctor blading technique can increase in-plane thermal conductivity using surface modified hexagonal BN (h-BN) (plate-like particle with ~10 μm diameter) with polydopamine (PDA) [56]. PDA treatment is worth noting because fillers are prevented from aggregating, while the dispersibility is enhanced in the poly(vinyl alcohol) (PVA) matrix during these processes (Figure 3b). Figure 3b shows cross-sectional SEM images of the aligned BN-PDA composite with 20% BN. Compared to the left image shown in Figure 3b, the right one is well aligned in the parallel direction (arrows) without aggregations. It has higher in-plane thermal conductivity than the casting method by as much as 13.5% at the same 20 vol % of BN concentration. Furthermore, doctor blading is an effective process to uniformly control film thickness so that composite films can have transmittance in a certain range of BN concentrations [61].

#### 3.2.3. Vacuum Assisted Assembly

Vacuum-assisted assembly is an attractive technique of forming densely packed composite with plate-like ceramics. When the dispersion of fillers in polymer or monomer solvent is subjected to a vacuum filtration system, the fluid is infiltrated into the pore of filter, aligning fillers in a parallel packed structure [65]. The higher the vacuum force, the higher the orientation and density of the composite film in the in-plane direction [43]. The vacuum-assisted assembly takes advantage of filtration to form densely aligned fillers in a small amount of matrix, resulting in high toughness and strength compared to other alignment techniques. Artificial nacre-like paper using non-covalent functionalized BN (a plate-like particle with a mean diameter of 200 nm) and a PVA matrix has been fabricated via a vacuum-assisted self-assembly technique [57]. Figure 3c shows a schematic of the preparation of composite paper with 6 wt % of PVA. SEM cross-sectional images of BN-PVA paper indicating a closely laminated structure of BN are shown in Figure 3c. The resulting composite with 94 wt % BN has a thermal conductivity of 6.9 W/mK. This method is suitable for achieving a high concentration of fillers in a composite since a polymer solution is filled into gaps between pre-filtered BN layers. This procedure does not need to have high dispersibility in the polymer matrix. Therefore, surface treatment is unnecessary, resulting in a simple process [65]. However, due to the stiffness of some flake-like fillers that cannot be easily bent but can crumble under external force, these fillers can damage each other and cause a fracture increase in the interface, especially for those with a large filler size and aspect ratio [66].

#### 3.2.4. Magnetic and Electric Field Alignment

Magnetic and electric field alignments are fascinating methods. Site-selective applied external magnetic and electric field can have a remote effect on fillers. They can control filler orientation and density inside the polymer matrix. They are powerful methods of allowing anisotropic fillers to have physical contact, thus inducing directional orientation toward the direction of external fields in order to minimize internal energy [67].

The magnetic field is affected in upward and downward rotations for anisotropic alignment of magnetite-treated fillers [32]. This method has been used to align ceramic fillers such as BN [52,58,68,69], SiC [69,70], and AlN [53]. For magnetic alignment, the modification of the surface of the filler is required because ceramics are not intrinsically magnetic materials. Figure 3d shows synthesized Fe_3_O_4_ on the surface of AlN (plate-like particle with a ~10 μm diameter) and the resulting AlN-Fe_3_O_4_ aligned in epoxy via sandwiching between two strong magnets [53]. Cross-sectional SEM images shown in Figure 3d reveal that the randomly dispersed filler has a non-uniform morphology. However, the magnetically aligned filler is dispersed and vertically arranged without filler aggregation. The thermal conductivity is enhanced by 92% compared to randomly dispersed composite with 20% AlN loading. Moreover, BN-Fe_3_O_4_/SiC-Fe_3_O_4_ binary filler has also been tried in order to increase heat transfer [68]. The addition of SiC nanoparticles functions as a thermal conducting bridge between BN particles. The enhancement of thermal conductivity in the through-plane direction is 162% with 40% loading compared to random composites. 

Electric field alignment is similar to magnetic force alignment from the perspective that fillers are indirectly affected by external force. However, electric field alignment relocalizes fillers and allows fillers to be simultaneously arranged linearly using DC electrical fields [71,72]. When an electric field is applied, anisotropic fillers like nanosheets and nanowires are polarized and the charge density of the longitudinal edge is increased, resulting in a field-induced torque acting on the sheet [73,74]. This promotes a parallel orientation of the anisotropic fillers and relocates the fillers for enhanced anisotropic thermal conductivity. Unlike magnetic field alignment, this technique enables untreated surface fillers to be used in arrangement [32]. However, it takes several hours to move the ceramic filler in the very viscous polymer matrix when using a magnetic or electric field to align the filler, which will limit their applications for practical uses.

### 3.3. Three-Dimensional (3D) Networked Assembly for Anisotropic Heat Conduction

As discussed above, effective alignment for the heat sinking layer is dependent on how to connect nanoceramic fillers through desired directions. Well-aligned nanoceramics that overcome the percolation threshold shown in Figure 2c can minimize heat resistance and enhance the heat conduction network. However, most alignments are successfully performed in a single, in-plane direction only, while vertical heat conduction is limited. Thus, building 3D networks in a polymer matrix has drawn attention in recent years to induce heat spreading through all or desired directions. In contrast to alignment on a plane, where fillers are connected in one direction, a 3D networking technique can achieve anisotropic fillers assemblies in a polymer matrix [33]. Here, we introduce these methods of constructing anisotropic paths of heat flow and enhancing the thermal conductivity of both in-plane and through-plane directions. Based on a comparison of these methods, we find that the thermal transfer efficiency per unit mass strongly depends on the geometry (3D > 2D > 1D > 0D) because the total interfacial area decreases as the filler dimension increases [43,75,76].

#### 3.3.1. Hot Pressing

**Figure 4:**
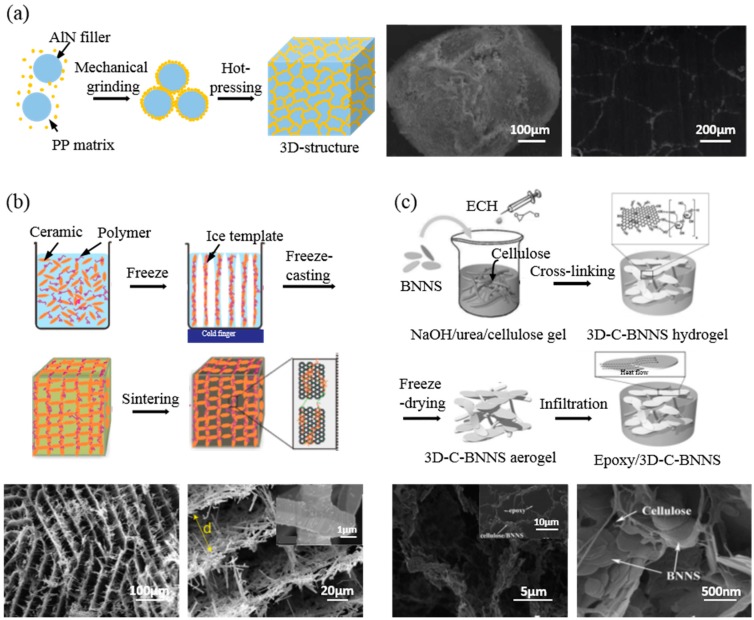
Schematic illustration of 3D networking of ceramic fillers/polymer composites and SEM images. (**a**) Schematic illustration of AlN/PP composite with a 3D segregated structure formed by hot pressing. SEM image of AlN-coated PP (10 vol % AlN) (left) and cross-sectional image of an AlN/PP 3D segregated composite (right) [8] are shown. Reprinted with permission from [8] Copyright (2015) Elsevier. (**b**) Schematic illustration of the preparation of 3D networking ceramic aerogel by freeze-casting. SEM images of honeycomb-like SiC network aerogel after sintering in different magnifications, and image of interconnected SiC by sintering (inset in bottom right) [45,77] are shown. {(**b**)(upper): Reprinted with permission from [45] Copyright (2015) John Wiley and Sons. (**b**)(bottom): Reprinted with permission from [77] Copyright (2016) John Wiley and Sons}. (**c**) Schematic illustration of the preparation of BN/cellulose/epoxy 3D network formed by self-assembly of nanoceramics. Cross-sectional SEM images of BN/cellulose networked aerogel at different magnifications and of a BN/cellulose/epoxy 3D network composite (inset in bottom left) [4]. Reprinted with permission from [4] Copyright (2016) John Wiley and Sons.

Hot pressing is a unique process that mixes inorganic powders at high pressure to produce ceramic-polymer composites. The mixture of polymer particles and ceramic filler particles produces a unique dispersion state in which the particle-shaped polymer is surrounded by the ceramic filler, which is termed a “core-shell structure” [8]. The mixture is then heat-treated under high pressure. It forms continuous filler chains around the polymer matrix [3]. Hu et al. fabricated a 3D segregated structure polypropylene (PP) particle core and an AlN shell using PP particles with sizes ranging from 300 to 500 μm and spherical AlN particles with sizes of up to 5 μm through mechanical grinding followed by sintering at 1.6 tons and 190 °C (Figure 4a). Figure 4a presents SEM images of PP-AlN particles and the composite. Fillers are localized selectively at the interface of PP particles. In this case, the thermal conductivity of the 30% AlN composite is 0.81 W/mK, which is 23.2% higher than that of the randomly dispersed composite by solution mixing [8]. This method also shows a different heat transfer property, depending on the size of the polymer particle. Different particle sizes induce different interfacial areas per unit volume at the same filler fraction. With increasing polymer particle size, the interface area between the fillers and the matrix is decreased. Therefore, the 3D network can be easily connected. By using larger-sized polymer particles, the thermal conductivity increases due to the stability of the thermal flow path in the composite [22]. In the hot pressing process, internal bubbles usually form between particles. Since pores induced by these bubbles can interfere with the heat transfer of the composite, they should be removed.

#### 3.3.2. Freeze-Casting

The freeze-casting method is based on a directional freezing technique. As the aqueous dispersion of fillers and polymer cools down, the growth of ice crystals expels fillers from the solid, constructing links of fillers. After the sintering process, fillers are compactly interconnected. After removing the ice crystal, a network structure is obtained without collapsing [45,76]. Ordered honeycomb-like networks using an aqueous suspension with SiC fibers (diameter ≈ 1.5 μm, length ≈ 20 μm), and PVA can be assembled [77]. Through anisotropic freezing and sintering, light and highly porous SiC structures exhibiting high strength (up to 3 MPa) and stiffness (up to 0.3 GPa) on a macroscopic scale are fabricated. Figure 4b shows SEM images of the SiC aerogel structure in a perpendicular direction toward the ice growth. Interestingly, these fillers are more densely interconnected in the through-plane direction than in the in-plane direction. This means that the composite has higher thermal conductivity in the perpendicular direction (0.65 W/mK) than in the parallel direction (0.54 W/mK). As shown in Figure 4b, the width between adjacent SiC walls is ordered. However, the direction is not in parallel. This phenomenon also appears in other structures. Zeng et al. have fabricated BN aerogel structures by changing the filler fraction. They found that an increasing proportion of fillers reduced the width between walls, and the direction of BN walls became more obviously perpendicular [45].

#### 3.3.3. Self-Assembly

To elucidate three-dimensional pathways for directional heat flow, chemical techniques such as self-assembly can be used by surface modifying fillers with functional groups. Reactive functional groups at the surface of fillers can combine fillers or structural templates, making an anisotropic network in the dispersed state [40]. However, in nondirectional linking, filler aggregation can occur, which prevents directional heat flow in the filler network [4]. Nanofibrillary cellulose structures can be used as a template to reduce filler aggregation and initiate directional networking without using external force such as shear stress or electromagnetic force. In addition, filler alignment can be controlled in a high concentration of filler suspension. A networked structure of nanofibrillary cellulose acts as a skeleton of filler alignment, to which a surface functionalized filler can attach by chemical interaction. Figure 4c illustrates a chemical technique to control the assembly of fillers. It is a kind of self-assembly technique to fabricate a 3D network. A 3D skeleton structure is constructed using cellulose nanofiber via sol-gel and freeze-drying first. Then, the BN filler (average diameter of 3 μm) is attached to the skeleton by cross-linking between fillers and skeletons. Epoxy resin is then added at the end of the process [4]. Such nanocomposite shows significant thermal conductivity enhancement, with 112% (‖) and 152% (⊥) enhancement at 9.29 vol % loading (Table 2). 

## 4. Application

Heat dissipation has been attractive in many kinds of applications such as flexible electronics, displays, and bio-integrated and implantable sensors [78,79,80,81,82]. Aligning techniques can be sure to induce high thermal conductivity, electrical insulation, a light weight, and a low thermal expansion coefficient in a low loading concentration of fillers. Therefore, ceramics composites with networked fillers are promising for use as substrates, TIM [83], and packages [84] of electronic devices.

**Figure 5:**
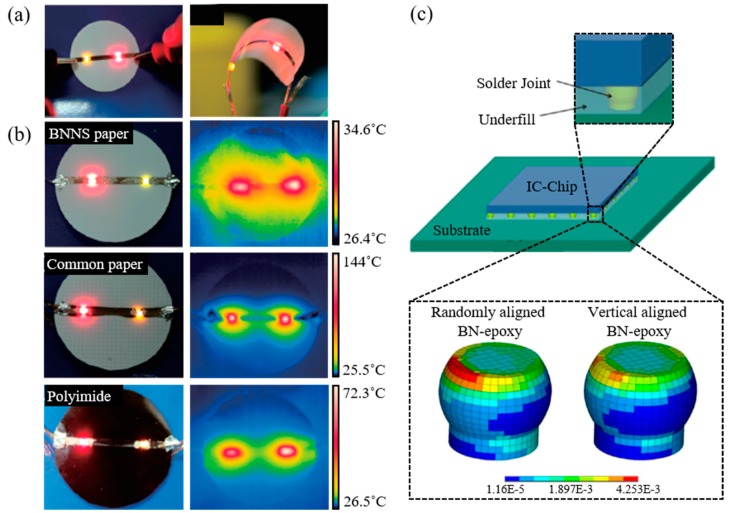
Practical examples of enhanced heat dissipation and minimized thermal shrinkage with aligned ceramic/polymer composites. (**a**,**b**) Heat dissipation results of LEDs on BN-PVA composite paper (upper), common paper (middle), and polyimide (bottom), depending on the substrate [57]. Reprinted with permission from [57] Copyright (2015) Royal Society of Chemistry. (**c**) Schematic illustration of an integrated IC chip with underfill using a BN-epoxy composite for encapsulation (upper). The simulation of an accumulated plastic strain to solder joints influenced by the underfill’s CTE of randomly dispersed and vertically aligned BN-epoxy composites (bottom) [52]. Reprinted with permission from [52] Copyright (2013) American Chemical Society.

Zeng et al. fabricated BN-PVA composite paper with flexibility in a low matrix fraction (6 wt % PVA) by vacuum-assisted self-assembly. Figure 5a shows performance during bending. Figure 5b shows outstanding heat dissipation compared to paper and polyimide substrates when a light-emitting diode (LED) is utilized. In the case of the LED, external quantum efficiency (EQE) and light output power are increased when temperature is decreased [85]. As a result, a composite with aligned fillers can improve the performance and reliability of LEDs. 

Aligning techniques can be used to fabricate TIM as an adhesive to connect a device to a substrate or to act as an underfill to fill void space of a solder joint between the device and the substrate. The TIM of a randomly dispersed ceramic composite has relatively low thermal conductivity (approximately 0.2–1.2 W/mK), while metal or carbon composite has high thermal conductivity (approximately 3–5 W/mK). However, such TIM requires an additional layer for electrical insulation [86]. Therefore, an aligned ceramic filler composite is a suitable candidate of TIM for overcoming these limitations. In addition to heat dissipation, aligned fillers can be used as underfill to minimize the coefficient of thermal expansion (CTE). Lin et al. have used magnetically aligned magnetite-coated BN-epoxy composite to reduce vertical CTE so as to diminish thermo-mechanical strain to the solder joint, thus improving the mechanical reliability of the solder joint interconnection between the substrate and IC chip. Figure 5c shows an assembled IC chip with solder joints and an underfilled BN-epoxy composite on an organic substrate to compare the effect of underfilled composites to solder joints depending on the filler alignment in the composites. The simulation of thermo-mechanical strains to the solder joint is conducted by finite elements analysis (FEA) simulated with a thermal cycle between 0 and 100 °C. The corner of the solder joint with a randomly aligned BN composite has the highest thermo-mechanical strain due to the z-direction CTE of the composite during the temperature cycle. The vertically aligned BN composite was found to be able to lower accumulated plastic strain on the solder joint. The simulation presents that vertically aligned fillers, compared to a randomly dispersed filler composite, can lower the z-direction CTE of the composite [52].

High thermal conductivity, low CTE, and electrical insulation are critical to thermal management of electronic devices [2]. An aligned nanoceramic composite is a promising candidate for single chip packaging and 3D chip packaging that requires electrical insulation and anisotropic heat flow. In the case of a vertically stacked 3D chip, the farther from the substrate that acts as a heat sink, the higher the temperature of the chip. One of the reasons is because glue layers and insulating layers between chips have low thermal conductivity [86]. By using an aligned composite as a glue and insulator layer, stacked heat in the upper chip layers can dissipate anisotropically to the heat sink.

## 5. Conclusions

Due to their high thermal conductivity and insulation properties, nanoceramics are fascinating materials. They are normally used as ceramic-polymer composites to dissipate heat from high-performance electronic devices to retain lifetime while increasing the reliability and performance of devices. Recently, increasing the thermal conductivity of ceramic composites with a relatively low fraction of fillers while flowing heat in a desired direction has also been investigated. In this paper, we summarized some ceramics that are normally used for high thermal conductivity, alignment methods of anisotropic ceramic fillers in the polymer matrix, and applications of aligned filler-based composites. 

As most research so far has been focused on enhancing heat spreading through a single in-plane direction, realization of vertical alignment formation for vertical heat conduction is very limited. Despite recent research studies on 3D networked assemblies we summarized here, the thermal conductivity of composites in the vertical direction is still lower than that in the in-plane direction. In addition, anisotropic heat transfer through 3D assembled nanofillers might have great potential for many applications. Nonetheless, the mass production of composite films with aligned fillers remains a challenge. We believe that more research studies are needed to achieve higher heat flow in the vertical direction, to load a high fraction of filler, and to overcome the limitations of time and scale.

## Figures and Tables

**Table 1:** Summary of thermal conductivity, dielectric constant, and electrical resistivity of nanoscale ceramic fillers at room temperature.

Ceramics		Thermal conductivity [W/mK]	Dielectric constant (at 1 MHz)	Electrical resistivity [Ω cm]	Ref.
AlN	Spherical	200–320	8.5–8.9	>10^14^	[28,33,34]
Al_2_O_3_	Spherical	30–42	6.0–9.0	>10^14^	[23,35,36]
SiC	Spherical	85–390	-	-	[33,36,37]
	Nanowire	90	40	-	[28]
Si_3_N_4_	Spherical	86–155	8.3	>10^13^	[3,38,39]
BN	Nanosheet	29–600	4.5	>10^13^	[32,33]
	Nanotube	200–300	-	-	[1]

**Table 2:** Summary of thermal conductivity at room temperature and enhancement factor depending on the ceramics, the matrix, and the alignment method (‖ and ⊥ are values of the in-plane and through-plane directions, respectively).

Ceramics	Matrix (Alignment method)	Loading [vol %]	Thermal conductivity [W/mK]	Enhancement factor/Solution mixing [%]	Ref.
AlN	PP (3D/Hot pressing)	30	0.81	23.2	[8]
		10	0.37		
	Epoxy/Fe_3_O_4_ (2D/Magnetic force)	20	1.754	92	[53]
Al_2_O_3_	PE-*g*-AA/HDPE (2D/Molding injection)	50 wt %	0.47 (‖) 0.305 (⊥)	17.5 (‖) −15.3 (⊥)	[23]
SiC	SiC fibre/air (3D/Ice template)	6	0.54 (‖) 0.65 (⊥)	-	[77]
	BN_Fe_3_O_4_/Epoxy (2D/Magnetic force)	40	5.77 (⊥) 2.25 (‖)	162 (⊥) −34.8 (‖)	[68]
Si_3_N_4_	PE (3D/Hot pressing)	20	1.20 (0.2 μm) 1.05 (3.0 μm) 0.94 (35 μm)	-	[39]
BN	PVA (2D/Vacuum assisted)	94 wt %	6.9	-	[57]
	Epoxy (3D/Fiber assisted self-assembly)	9.6	3.13	998	[4]
	PVA/PVA_PDA (2D/Doctor blading)	30	7.27 (‖) 8.8 (‖)	21.2 (‖) 13.5 (‖)	[56]
	Epoxy (2D/Magnetic force)	20 wt %	0.85 (‖)	104 (⊥)	[52]
	PVA (3D/Ice template)	9.29	2.85 (‖) 2.40 (⊥)	112 (‖) 152 (⊥)	[45]
